# Live birth/parity number and the risk of incident hypertension among parous women during over 13 years of follow‐up

**DOI:** 10.1111/jch.14369

**Published:** 2021-10-17

**Authors:** Seyyed Saeed Moazzeni, Samaneh Asgari, Fereidoun Azizi, Farzad Hadaegh

**Affiliations:** ^1^ Prevention of Metabolic Disorders Research Center Research Institute for Endocrine Sciences Shahid Beheshti University of Medical Sciences Tehran Iran; ^2^ Endocrine Research Center Research Institute for Endocrine Sciences Shahid Beheshti University of Medical Sciences Tehran Iran

**Keywords:** incident hypertension, live birth, parity, Tehran Lipid and Glucose Study

## Abstract

The effect of live birth/parity number on incident hypertension was investigated among Iranian parous women aged 30–70 years. The study population included 2188 normotensive women who were enrolled in 1999–2001. They were followed for incident hypertension (based on JNC 7 report) by 3‐year intervals up to April 2018. Multivariable Cox proportional hazard models, adjusted for a wide set of potential hypertension risk factors, reproductive factors, and pregnancy complications, were applied to estimate hazard ratios (HRs) and 95% confidence intervals (CIs) of the number of parity/live birth(s) for incident hypertension. Additionally, as a sensitivity analysis, age‐scale Cox regression was also done. During a median follow‐up of 13.5 years, 935 incident hypertension have occurred. Compared to those with two live births, the participants who had 3 and ≥4 live births were at higher risk of hypertension development by the HRs of 1.25 [95% CI: 1.02–1.55] and 1.39 [1.12–1.72], respectively, in the full‐adjusted model. Moreover, each additional live birth increased the risk of hypertension by a HR of 1.06 [95%CI: 1.02–1.11]. Results of parity number were also similar. Considering age as time scale also did not change the results generally. The authors found a significant interaction between live birth/parity number and age groups; the adverse effect of higher live birth/parity numbers on hypertension development was mainly found among those aged < 50 years. To sum up, compared to the live birth/parity number of two, Iranian women with ≥3 live birth/parity had a higher risk of incident hypertension; the issue was more prominent among younger mothers.

## 1 INTRODUCTION

Hypertension, the leading cause of cardiovascular disease (CVD) and premature death, was prevalent in 31.1% of adults (1.39 billion) worldwide. Most of these hypertensive cases live in low‐ and middle‐income countries.^1^ From 2005 to 2011 in Iran, due to improvements in awareness, management, treatment, and control of hypertension, there is a gradual decline in the prevalence; however, in 2011, a quarter of Iranian adults aged 30–70 years still were hypertensive.^2^ We also previously reported that about 2.7% of Tehranian adults (2.6% for women) developed hypertension annually.^3^

In addition to known hypertension risk factors such as aging, dietary factors (high sodium intake, low potassium intake, and unhealthy diet), obesity, lack of physical activity, and other potential hypertension risk factors,^1^ childbearing and some other reproductive factors were suggested to have an association with blood pressure (BP) level and hypertension among women.^4^, ^5^, ^6^ Physiological changes during pregnancy, labor, and also postpartum periods influence the BP levels.^7^ Moreover, physiologic cardiometabolic changes (weight gain, insulin resistance, increased plasma glucose, and dyslipidemia) and complications that women might experience during pregnancy exert potential short‐ and long‐term effects on postpartum outcomes, especially cardiovascular ones.^8^, ^9^, ^10^, ^11^, ^12^, ^13^

Most of the previous findings of the effect of live birth and parity on BP level or hypertension were limited to cross‐sectional studies with inconsistent results. Although some previous studies suggested that parity can have lowering effects on BP level,^14^, ^15^ many more have reported that as the live birth or parity number increased, the prevalence of hypertension became higher.^16^, ^17^, ^18^ Moreover, findings from prospective studies on this issue, all conducted in Western countries, were also limited and inconclusive.^6^, ^19^, ^20^, ^21^

The current study aims at determining whether the live birth/parity number is an independent risk factor for hypertension development among Iranian women aged 30–70 years, with 13.5 years of follow‐up, using a population‐based cohort, the Tehran Lipid and Glucose Study (TLGS).

## 2 MATERIALS AND METHODS

### 2.1 Study design and study population

The TLGS is a prospective cohort study. This study was conducted on a representative sample of Tehranian citizens that resided in district 13 of Tehran. Determining the prevalence, incidence, and other epidemiologic aspects of non‐communicable diseases (NCDs) and also prevention of NCDs by advancing healthier lifestyles were the original aims of the TLGS. The details of the design, measurement methods, and enrollment strategy of the TLGS have been described elsewhere.^22^ To summarize, the first phase of the TLGS, which was on January 31, 1999–July 3, 2001, is considered as enrollment. Data collection for prospective follow‐up phases were repeated up for at least 20 years with approximately 3‐year intervals (ie, phase II: 2001–2005, phase III: 2005–2008, phase IV: 2008–2011, phase V: 2011–2014, and phase VI: 2015–2018).

Among a total of 4283 female participants aged 30–70 years, 153 single women, and eight married women with no live birth were excluded. We also excluded 1212 patients with prevalent hypertension and 70 patients with prevalent CVD at baseline, leading to 2840 women. Other reasons for exclusion were missing data on parity/live birth number and other covariates (*n* = 243), and no follow‐up measurement (*n* = 409), remaining 2188 eligible women to follow‐up to April 2018.

The ethics committee of the Research Institute for Endocrine Sciences of Shahid Beheshti University of Medical Sciences approved the current study proposal. Written informed consent was obtained from all participants. Moreover, all methods and measurements were done following the relevant guidelines and regulations.

### 2.2 Clinical and laboratory measurements

According to the TLGS protocol,^22^ at each visit, we used interviewer‐administered questionnaires to obtain demographic data, past medical and drug history, family history of premature CVD, marital status, and smoking habits. Moreover, the interviewer asked female participants their menopausal status, their history of miscarriage, and the live birth/parity number that they had.

We measured weight by a digital scale to the nearest 100 g and height in a standing position while participants had light clothing and no shoes. Body mass index (BMI) was calculated as weight in kilograms divided by the square of height in meters. We also measured waist circumference (WC) at the level of the umbilicus with light clothing. Subsequent to 15 min of rest, using a standardized mercury sphygmomanometer (calibrated by the Iranian Institute of Standards and Industrial Research), BP measured two times by a trained physician on the right arm in a sitting position in the TLGS office. The mean of these two office BP measurements was considered as the patient's BP. After at least 12 h of fasting, morning blood samples were collected from all participants. Measurements of fasting plasma glucose (FPG), triglycerides (TG), and high‐density lipoprotein cholesterol (HDL‐C) were performed by standard and validated methods, as explained before.^22^

### 2.3 Definition of main exposure, confounding factors, and outcome

Main exposure: The sum of the number of live birth and the stillbirth (defined as the birth of an infant which died in the mother's uterus after 20 weeks of gestation) was considered as parity number.

Confounder factors: Diabetes mellitus (DM) was defined as a FPG level of ≥7.0 mmol/L or pharmacologically treated with glucose‐lowering drugs. Smoking status was categorized into two groups; current smokers versus former/never smokers. Prior diagnosed CVD in female first‐degree blood relatives aged < 65 years or male first‐degree blood relatives aged < 55 years was defined as a positive family history of premature CVD. Based on the World Health Organization's definition, the absence of spontaneous menstrual bleeding for more than 12 months, without other pathologic or physiologic cause could be determined as menopause^23^; for participants with missing information, the menopausal age was considered at ≥50 years.^24^ Based on the standard definition of the onset of a BP level ≥140/90 mm Hg with proteinuria > 0.3 g/24 h after 20 weeks’ gestation,^25^ preeclampsia diagnosis was made as a part of routine maternal care in Iran. At the enrollment phase, using a validated self‐reporting questionnaire,^26^ female participants were asked about their history of preeclampsia.^27^ Finally, gestational DM (GDM) was defined as the presence of macrosomia or a history of GDM that self‐reported by participants.^28^ Macrosomia was considered as a birth weight > 4 kg.^29^

Outcome: Based on the seventh report of the joint national committee on prevention, detection, evaluation, and treatment of high BP (the JNC 7 report), hypertension, whether at baseline or as an outcome during follow‐up, was defined as the presence of at least one of the following criteria: (a) having SBP≥140 mm Hg, (b) having DBP≥90 mm Hg, and (c) initiation of anti‐hypertensive drugs usage. Moreover, SBP 120–139 mm Hg or DBP 80–89 mm Hg were defined as prehypertension.^30^

### 2.4 Statistical analyses

Descriptive statistics were used to describe baseline characteristics as mean ± standard deviation (SD) for normally distributed continuous variables, median (interquartile range: IQR) for the highly skewed variables, and number (%) for categorical variables. We compared the baseline characteristics of the participants across the number of live birth (1, 2, 3, and ≥4) using chi‐square, fisher's exact, ANOVA, and Kruskal–Wallis tests as appropriate.

The time to event was described as the time of censoring or the outcome occurring, whichever firstly came. In the case of death, leaving the district, or being until the end of study phase VI (April 2018) without any event, we censored participants. For individuals with incident hypertension, the event date was defined as the mid‐time between the dates of the follow‐up visit at which a patient developed hypertension and the last follow‐up visit preceding incident hypertension.

Cox proportional hazard models were used to evaluate the association of parity and live birth number with incident hypertension. Parity and live birth number were considered as both continuous and categorical variables in the Cox models, separately. We considered live birth/parity number of two as the reference group, given that this number was associated with the lowest risk for CVD event among Iranian women.^13^ In the current study, the selection of confounders was derived from our recent study among the Iranian female population.^3^ It should be noted that we used the baseline data of the covariates. The hazard ratios (HRs) with 95% confidence intervals (CIs) were reported in four models: Model 1: adjusted for age; Model 2: adjusted for age, BMI, WC, DM, family history of premature CVD, current smoking, TG/HDL‐C, menopausal status, and oral contraceptive pill (OCP) usage; Model 3: Model 2 + further adjusted for preeclampsia and GDM; Model 4: Model 3 + further adjusted for prehypertension. Additionally, as a sensitivity analysis, age‐scale Cox regression was also done.

Since the effect of parity/live birth number on BP might be differed by menopausal status^15^, ^18^ and aging,^31^ hence we tested the interaction of parity/live birth number with menopausal status (premenopausal versus postmenopausal) and also age‐groups (<50 versus ≥50 years old) in the fully adjusted model 4. Because of significant interaction between age groups and live birth/parity number (all *p*‐values for interaction were lower than .02), HRs with 95% CIs were reported for each subgroup of age separately.

Using the Schoenfeld residual test, the proportionality was assessed for the Cox models. All proportionality assumptions were appropriate in this study. STATA version 14 (StataCorp LP, College Station, TX, USA) statistical software was used for statistical analyses of the current study and a two‐tailed *p*‐value ≤.05 was considered significant.

## 3 RESULTS

The study population consists of 2188 female participants with a mean age (SD) of 43.64 (9.53) years. The baseline characteristics of the study population according to the number of live birth are presented in Table 1. In general, by increasing in the number of live birth, cardiometabolic risk profiles were worse among continuous variables except for HDL‐C; participants with ≥4 live birth had older age, higher levels of BMI, WC, BP, FPG, TG, and TG/HDL‐C. Among categorical variables, although who had ≥4 live births took OCP less than other groups, using glucose‐lowering drugs and being postmenopausal were more prevalent among them. DM prevalence also increased as the number of live birth increased. Furthermore, women with ≥4 live births had higher and lower prevalence of history of GDM and preeclampsia, respectively.

**TABLE 1:** Baseline characteristics according to the number of live birth: Tehran Lipid and Glucose Study, Iran, 1999–2018

Number of live birth	1	2	3	≥4	*p*‐value^a^	Total population
Number of participants	189	583	563	853		2188
Continuous variables, Mean (SD)						
Age (year)	36.78 (8.43)	37.75 (6.21)	42.10 (7.96)	50.20 (8.41)	< .001	43.64 (9.53)
BMI (kg/m^2^)	26.54 (4.31)	27.24 (4.29)	28.07 (4.31)	28.94 (4.44)	< .001	28.06 (4.43)
WC (cm)	84.33 (10.64)	85.10 (10.34)	88.46 (10.92)	92.68 (11.00)	< .001	88.85 (11.28)
SBP (mm Hg)	109.83 (11.57)	109.92 (10.34)	113.00 (11.78)	116.41 (11.29)	< .001	113.24 (11.54)
DBP (mm Hg)	73.95 (8.19)	74.68 (7.46)	75.84 (7.56)	76.22 (7.26)	< .001	75.51 (7.51)
FPG (mmol/L)	5.08 (1.41)	5.06 (1.23)	5.17 (1.32)	5.81 (2.37)	< .001	5.38 (1.83)
HDL‐C (mmol/L)	1.18 (0.26)	1.16 (0.27)	1.15 (0.28)	1.15 (0.30)	.413	1.15 (0.28)
TG (mmol/L)	1.28 (0.95)	1.39 (1.04)	1.62 (1.16)	1.81 (1.32)	< .001	1.59 (1.18)
Categorical variables, number (%)						
Family history of premature CVD, yes	39 (20.6%)	108 (18.5%)	92 (16.3%)	149 (17.5%)	.546	388 (17.7%)
Current smoking, yes	12 (6.3%)	38 (6.5%)	24 (4.3%)	40 (4.7%)	.261	114 (5.2%)
Glucose‐lowering drugs, yes	2 (1.1%)	8 (1.4%)	7 (1.2%)	57 (6.7%)	< .001	74 (3.4%)
Diabetes mellitus, yes	4 (2.1%)	17 (2.9%)	24 (4.3%)	103 (12.1%)	< .001	96 (14.7%)
Menopause, yes	17 (9.0%)	45 (7.7%)	118 (21.0%)	415 (48.7%)	< .001	595 (27.2%)
OCP use, yes	16 (8.5%)	57 (9.8%)	49 (8.7%)	25 (2.9%)	< .001	147 (6.7%)
History of preeclampsia, yes	13 (6.9%)	50 (8.6%)	39 (6.9%)	37 (4.3%)	.011	139 (6.4%)
History of GDM, yes	3 (1.6%)	35 (6.0%)	61 (10.8%)	128 (15.0%)	< .001	227 (10.4%)

Values are shown as Mean (SD) and number (%) for continuous and categorical variables, respectively; values are shown as Median (interquartile range) for TG.

*Abbreviations*: SD, Standard deviation; BMI, body mass index; WC, waist circumference; SBP, systolic blood pressure; DBP, diastolic blood pressure; FPG, fasting plasma glucose; TG, triglycerides; HDL‐C, high density lipoprotein cholesterol; OCP, oral contraceptive pill; GDM, gestational diabetes mellitus.

^a^The comparison p‐value between groups was calculated using ANOVA test for normal continues variables, Kruskal–Wallis test for skewed variables and chi‐square test (fisher's exact test if required) for categorical variables.

**FIGURE 1:**
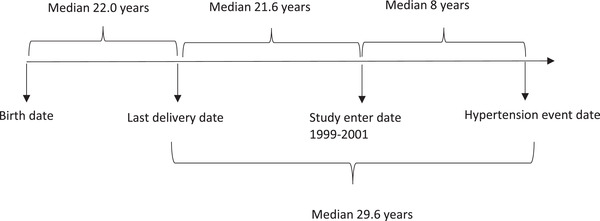
Timeline chart for participants who developed hypertension during follow‐up (*n* = 935): Tehran Lipid and Glucose Study, Iran, 1999–2018

During a median follow‐up of 13.58 years (IQR:7.36–15.94), 935 incident hypertension have occurred. As shown in Figure 1, for participants developed hypertension during follow‐up, the median interval between last labor and incident hypertension is 29.6 years, that stratified to 21.6 and 8 years before and after recruitment of the study, respectively. Compared to those with two live births, the participants who had three, four, and over children were at a higher age‐adjusted risk of incident hypertension. Having 3 and ≥4 live births had significant risk after further adjustment for traditional and reproductive factors (menopausal status and OCP use) in model 2, and preeclampsia and GDM in model 3. Even in model 4, which was further adjusted for prehypertension, the corresponding HRs remained significant (1.25 [95%CI:1.02–1.55] for three live births and 1.39 [1.12–1.72] for ≥4 live births). Importantly, in our data analysis, traditional hypertension risk factors, including older age, higher BMI, DM, positive family history of premature CVD, prehypertension, GDM (marginally significant), and preeclampsia, were associated with incident hypertension (Table 2). Results for parity number were also similar; in comparison with parity number of 2, having 3 or ≥4 parity increased the risk of incident hypertension by the HRs of 1.25 [1.01–1.54] and 1.40 [1.13–1.74] in model 4, respectively (Table S1). Moreover, each additional live birth and parity (considering as continuous variables) increased the risk of incident hypertension significantly in model 4 (1.06 [1.02–1.11] for live birth number and 1.06 [1.02–1.10] for parity number) (Table 3 and Table S2).

**TABLE 2:** Multivariable hazard ratios (HR) and 95% confidence intervals (CI) of incident hypertension according to the number of live birth among women: Tehran Lipid and Glucose Study, Iran, 1999–2018

	Model 1		Model 2		Model 3		Model 4	
	HR (95% CI)	*p*‐value	HR (95% CI)	*p*‐value	HR (95% CI)	*p*‐value	HR (95% CI)	*p*‐value
Number of live birth								
1	1.00 (0.72–1.38)	.996	1.00 (0.72–1.38)	.991	1.00 (0.73–1.39)	.985	0.97 (0.70–1.35)	.868
2	Reference		Reference		Reference		Reference	
3	1.38 (1.12–1.70)	.002	1.31 (1.07–1.62)	.011	1.30 (1.06–1.60)	.013	1.25 (1.02–1.55)	.035
≥ 4	1.66 (1.35–2.05)	<.001	1.42 (1.15–1.75)	.001	1.40 (1.13–1.72)	.002	1.39 (1.12–1.72)	.003
Age (year)	1.05 (1.04–1.06)	<.001	1.04 (1.03–1.06)	<.001	1.05 (1.03–1.06)	<.001	1.04 (1.03–1.05)	<.001
BMI (kg/m^2^)			1.03 (1.01–1.06)	.010	1.03 (1.01—1.06)	.013	1.03 (1.00–1.05)	.036
WC (cm)			1.01 (1.00–1.02)	.018	1.01 (1.00–1.02)	.024	1.01 (1.00–1.02)	.139
DM			1.80 (1.46–2.22)	<.001	1.72 (1.39–2.13)	<.001	1.61 (1.30–1.99)	<.001
Family history of premature CVD			1.23 (1.05–1.45)	.010	1.22 (1.04–1.44)	.014	1.20 (1.02–1.41)	.025
Current smoking			0.91 (0.66–1.26)	.580	0.91 (0.66–1.25)	.546	0.97 (0.70–1.33)	.840
TG/HDL‐C			1.01 (1.00–1.03)	.143	1.01 (1.00–1.03)	.126	1.01 (1.00–1.03)	.133
Menopause			1.03 (0.84–1.27)	.771	1.04 (0.84–1.27)	.740	1.08 (0.87–1.32)	.491
OCP use			1.05 (0.78–1.42)	.745	1.04 (0.77–1.41)	.804	1.02 (0.76–1.38)	.881
History of preeclampsia					1.36 (1.04–1.76)	.022	1.32 (1.02–1.72)	.035
History of GDM					1.18 (0.97–1.42)	.097	1.20 (0.99–1.45)	.060
Prehypertension							2.30 (1.99–2.65)	<.001

*Abbreviations*: BMI, body mass index; WC, waist circumference; DM, diabetes mellitus; CVD, cardiovascular disease; FH, family history; TG, triglycerides; HDL‐C, high‐density lipoprotein cholesterol; OCP, oral contraceptive pill; GDM, gestational diabetes mellitus.

Model 1: Adjusted for age.

Model 2: Adjusted for age, BMI, WC, DM, family history of premature CVD, current smoking, TG/HDL‐C, menopausal status, and OCP use.

Model 3: Model 2 + further adjusted for preeclampsia and GDM.

Model 4: Model 3 + further adjusted for prehypertension.

**TABLE 3:** Multivariable hazard ratios (HR) and 95% confidence intervals (CI) of incident hypertension per additional live birth among women: Tehran Lipid and Glucose Study, Iran, 1999–2018

	Model 1		Model 2		Model 3		Model 4	
	HR (95% CI)	*p*‐value	HR (95% CI)	*p*‐value	HR (95% CI)	*p*‐value	HR (95% CI)	*p*‐value
Each additional live birth	1.09 (1.05–1.13)	<.001	1.06 (1.02–1.11)	.003	1.06 (1.02–1.11)	.004	1.06 (1.02–1.11)	.003
Age (year)	1.05 (1.04–1.06)	<.001	1.04 (1.03–1.06)	<.001	1.05 (1.03–1.06)	<.001	1.04 (1.03–1.05)	<.001
BMI (kg/m^2^)			1.03 (1.01–1.06)	.008	1.03 (1.01–1.06)	.010	1.03 (1.00–1.05)	.031
WC (cm)			1.01 (1.00–1.02)	.014	1.01 (1.00–1.02)	.021	1.01 (1.00–1.02)	.123
DM			1.77 (1.44–2.18)	<.001	1.69 (1.37–2.09)	<.001	1.59 (1.28–1.96)	<.001
Family history of premature CVD			1.24 (1.05–1.45)	.010	1.23 (1.04–1.44)	.013	1.21 (1.03–1.42)	.022
Current smoking			0.90 (0.65–1.23)	.495	0.89 (0.65–1.22)	.465	0.95 (0.69–1.31)	.754
TG/HDL‐C			1.01 (1.00–1.03)	.097	1.01 (1.00–1.03)	.086	1.01 (1.00–1.03)	.092
Menopause			1.02 (0.83–1.25)	.847	1.03 (0.83–1.26)	.808	1.06 (0.86–1.31)	.563
OCP use			1.05 (0.78–1.43)	.730	1.04 (0.77–1.41)	.798	1.02 (0.75–1.38)	.903
History of preeclampsia					1.36 (1.05–1.77)	.020	1.33 (1.03–1.73)	.031
History of GDM					1.19 (0.98–1.44)	.074	1.22 (1.01–1.47)	.043
Prehypertension							2.31 (2.00–2.66)	<.001

*Abbreviations*: BMI, body mass index; WC, waist circumference; DM, diabetes mellitus; CVD, cardiovascular disease; FH, family history; TG, triglycerides; HDL‐C, high‐density lipoprotein cholesterol; OCP, oral contraceptive pill; GDM, gestational diabetes mellitus.

Model 1: Adjusted for age.

Model 2: Adjusted for age, BMI, WC, DM, family history of premature CVD, current smoking, TG/HDL‐C, menopausal status, and OCP use.

Model 3: Model 2 + further adjusted for preeclampsia and GDM.

Model 4: Model 3 + further adjusted for prehypertension.

In a sensitivity analysis, considering age scale Cox regression, each additional live birth or parity was associated with a 4% higher risk of incident hypertension in model 4 (for parity, the risk was marginally significant). Moreover, after adjustment for a wide series of confounders in model 2, compared to the reference group (live birth/parity number = 2), having 3 and ≥4 live birth/parity(s) increased the risk significantly; even with the further adjustment of prehypertension, preeclampsia and GDM, the association remained marginally significant for live birth number of ≥4 (1.22 [0.99–1.50; *p*‐value: .056], and significant for parity number of ≥4 (1.24 [1.01–1.52]) in model 4 (Table 4).

**TABLE 4:** Multivariable hazard ratios (HR) and 95% confidence intervals (CI) of incident hypertension among women, using age as time scale: Tehran Lipid and Glucose Study, Iran, 1999–2018

	Model 1		Model 2		Model 3		Model 4	
	HR (95% CI)	*p*‐value	HR (95% CI)	*p*‐value	HR (95% CI)	*p*‐value	HR (95% CI)	*p*‐value
Each additional live birth	1.09 (1.05–1.13)	<.001	1.05 (1.01–1.09)	.016	1.05 (1.01–1.09)	.018	1.04 (1.00–1.08)	.041
Number of live birth								
1	1.08 (0.78–1.49)	.651	1.06 (0.76–1.46)	.742	1.06 (0.76–1.46)	.738	1.02 (0.74–1.42)	.884
2	Reference		Reference		Reference		Reference	
3	1.32 (1.07–1.62)	.009	1.24 (1.00–1.52)	.046	1.23 (1.00–1.51)	.051	1.17 (0.95–1.44)	.141
≥ 4	1.55 (1.27–1.89)	<.001	1.29 (1.05–1.58)	.014	1.28 (1.04–1.57)	.018	1.22 (0.99–1.50)	.056
Each additional parity	1.08 (1.04–1.12)	<.001	1.05 (1.01–1.09)	.022	1.05 (1.01–1.09)	.025	1.04 (1.00–1.08)	.055
Number of parity								
1	1.10 (0.79–1.54)	.581	1.07 (0.76–1.49)	.700	1.07 (0.76–1.49)	.703	1.06 (0.76–1.48)	.739
2	Reference		Reference		Reference		Reference	
3	1.31 (1.06–1.62)	.012	1.24 (1.00–1.53)	.050	1.23 (1.00–1.52)	.054	1.17 (0.94–1.44)	.152
≥ 4	1.57 (1.28–1.91)	<.001	1.30 (1.06–1.60)	.011	1.29 (1.05–1.58)	.015	1.24 (1.01–1.52)	.043

Model 1 is a crude model.

Model 2: Adjusted for body mass index, waist circumference, diabetes mellitus, family history of premature cardiovascular disease, current smoking, triglycerides/ high‐density lipoprotein cholesterol, menopausal status, and oral contraceptive pill (OCP) use.

Model 3: Model 2 + further adjusted for preeclampsia and gestational diabetes mellitus.

Model 4: Model 3 + further adjusted for prehypertension.

In our data set, 133 participants had another delivery during follow‐up. As another sensitivity analysis, we rerun the extended Cox regression by considering time‐varying analysis for live birth/ parity number; each additional live birth and parity (considering as continuous variables) increased the risk of incident hypertension significantly in model 4 (1.05 [1.01—1.10] for live birth number and 1.05 [1.01–1.09] for parity number). Moreover, after excluding these 133 participants, our results for women without having other delivery after baseline were similar to main analysis (Table S3).

**FIGURE 2:**
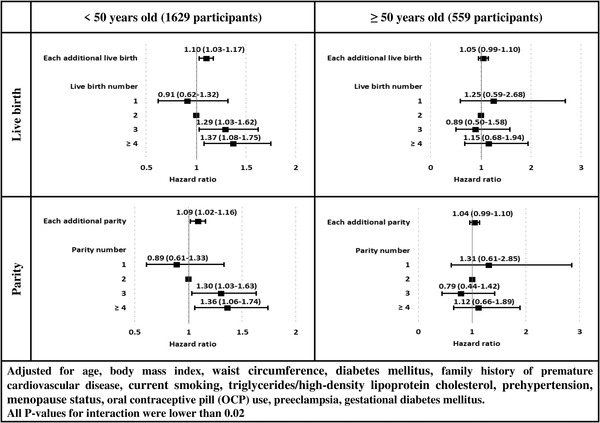
Multivariable hazard ratios (HR) and 95% confidence intervals (CI) of incident hypertension by live birth/parity number in different age‐groups: Tehran Lipid and Glucose Study, Iran, 1999–2018

The interaction of menopausal status and live birth/parity number (whether considering as continuous or categorical) was non‐significant (all *p*‐values > .05). On the other hand, live birth/parity number, whether as continuous or categorical variables, had a different effect on hypertension development in < 50‐year and ≥50‐year participants (all *p*‐values for interaction were lower than .02). Multivariable HRs and 95% CIs in different subgroups of age are shown in Figures 2. No significant association was found for those aged ≥50 years; however, among participants aged < 50 years, those with 3 and ≥4 of live birth/parity number were at higher risk of incident hypertension.

## 4 DISCUSSION

During more than 13.5 years of follow‐up, we found that higher live birth/parity numbers have an adverse impact on incident hypertension among Iranian mothers. After adjustment for a wide set of important confounders including age, BMI, WC, DM, family history of premature CVD, current smoking, TG/HDL‐C, prehypertension, menopausal status, OCP use, preeclampsia, and GDM, compared to those with two live births, participants with 3 and ≥4 live births were at 25 and 39% higher risk of hypertension development, respectively. Importantly, the interval between the last labor and hypertension development was about 30 years. Results for parity numbers were also similar to live birth number in our data analysis. Moreover, we found that the adverse effect of higher live birth/parity numbers on hypertension development was mainly found among those who were younger than 50 years old.

The associations of live birth/parity number with hypertension and BP levels have been examined in several previous cross‐sectional studies; however, their results were inconsistent. In a cross‐sectional study on African‐American women, Taylor and coworkers suggested that parity may increase the risk of hypertension through increased SBP and BMI, although increased parity and BMI may also serve as lowering factors for DBP.^32^ Similar to the Taylor and coworkers study, other cross sectional studies have shown that higher parity and live birth numbers were associated with higher prevalence of hypertension and BP levels in Mali,^33^ Turkey,^16^, ^17^ and Netherland,^12^ which were in line with our findings. Among Korean women, on the other hand, it was found that parity was inversely correlated with both SBP and DBP.^15^ Another cross‐sectional analysis from Bangladesh also showed that among women aged 18–75 years, compared to those with one parity, participants with ≥2 parity had higher levels of DBP and lower level of SBP.^34^ Moreover, in some other cross‐sectional studies, live birth/parity number did not significantly associate with BP levels and hypertension, or lose their significance after adjustment for confounders.^35^, ^36^, ^37^, ^38^

Based on a study on perimenopausal and postmenopausal women from Italy, Giubertoni and coworkers found that compared with nulliparous women, those with at least one child had higher prevalence of hypertension; however, the incidence of hypertension during follow‐up was not related with parity.^21^ Prospective data from US women who were parous at baseline showed that adjusted mean BP did not differ by the number of subsequent births; however, for those who were nulliparous at baseline, adjusted mean BP decreased by subsequent births.^6^ Results from the HUNT study in Norway also demonstrated that successive pregnancies were associated with lasting and clinically relevant reductions in SBP and DBP; it took about a decade for parous women to reach the levels they had experienced before pregnancy.^20^ In contrast to the studies mentioned above, among Danish premenopausal women, researchers found that as the number of live‐birth pregnancies increased, the risk of incident hypertension increased between live‐birth pregnancies during follow‐up.^19^ Similar to results from Denmark, we also found an unfavorable impact of higher numbers of live birth/parity among Iranian women; the issue was more prominent among younger ones. The non‐significant association among older participants may be due to greater contribution of aging and other traditional risk factors on hypertension development in the elderly, especially after menopause. Another possible reason is the limited number of older participants in our study. In contrast to our results, a population‐based study in Switzerland also demonstrated that parity had a decreasing and increasing effect on BP level in younger and older groups, respectively (*p*‐value for interaction < .001); however, due to cross‐sectional design of this study, their finding was not comparable with our prospective results.^31^

The observed higher hypertension risk and prevalence by increasing in live birth/parity number was suggested to be mainly attributed to the pathway of metabolic changes (weight gain, dyslipidemia, insulin resistance, and increased plasma glucose) that occur during pregnancy physiologically.^39^ By multiple pregnancies, the exposure time to these changes was increased. This accumulative effect of repeating parity can be associated with incident metabolic diseases such as metabolic syndrome, obesity, T2DM, and CVD in the future life of mothers,^9^, ^10^, ^11^, ^12^, ^40^, ^41^ and consequently, hypertension development, since these disorders are known hypertension risk factors. We suggest that although this pathway can have a role, but it could not justify the unfavorable impact of higher live birth/parity number completely, because in our data analysis, we adjusted for general and central obesity, DM, lipid profile, and also prehypertension as confounders and results remained significant. Moreover, even after considering the history of preeclampsia and GDM, which were extreme of these metabolic changes pathologically, results did not change. As another explanation, some human and animal studies suggested that multiple pregnancy can cause endothelial dysfunction and greater pressure response to vasoconstrictive agents.^42^, ^43^, ^44^, ^45^, ^46^, ^47^ In addition to the biological pathway, multiparity was found to develop CVD through an unhealthier lifestyle and socioeconomic factors.^8^, ^48^, ^49^ It was reported in Iran that household income per capita is lower in higher family size.^50^ Furthermore, at the recruitment time of this study, Iranian government had a policy of reducing population growth, so there was minimal economic support for Iranian parents.^51^ Therefore, mothers with more children were at higher economical pressure and have lower leisure time. It can lead to unhealthier diet (less access to high fiber and fruit rich diet), physical inactivity, poor access to health care, and other socioeconomic problems. Therefore, health policymakers need to pay special attention to education on health, nutrition, and special economic support of mothers willing to have more than two children.

The key strengths of this study are its long duration of follow‐up, standardized measurements for confounders and outcome rather than relying on self‐reported data, and using a wide set of confounders, especially complications of pregnancy that poorly addressed in previous studies. However, several important limitations need to be considered. First, no access exists for valid data on job status, income, and diet habits of participants. These factors can be the socioeconomic and lifestyle factors that have a potential effect on hypertension development. Second, the number of female participants with no children at baseline was too low (*n* = 8) to compare the impact of nulliparity with ever parity. Third, we relied on office BP measurements using non‐automated device rather than ambulatory or home BP measurements; hence, we cannot diagnose those with “white coat” hypertension or “masked” hypertension. However, the method applied in other population‐based studies.^6^, ^20^ Finally, all of our participants belonged to an urban area only, and our findings may be unable to be generalizable to rural populations.

To sum up, during more than a decade of follow‐up, among residents of the metropolitan city of Tehran, compared to live birth/parity number of two, those with ≥3 live birth/parity had higher risk of incident hypertension, independent of well‐known hypertension risk factors and reproductive factors; the issue was more prominent among younger mothers. Further investigations are needed to evaluate this issue in other parts of the country and also discover the potential role of socioeconomic and lifestyle factors in the pathway between live birth/parity and hypertension development.

## CONFLICTS OF INTEREST

There are no conflicts of interest.

## AUTHOR CONTRIBUTIONS

Study conception and design: Seyyed Saeed Moazzeni and Farzad Hadaegh; Analysis and interpretation of data: Samaneh Asgari and Farzad Hadaegh; Drafting of the manuscript: Seyyed Saeed Moazzeni and Farzad Hadaegh; Critical revision: Fereidoun Azizi and Farzad Hadaegh. All authors read and approved the final manuscript.

## Supporting information

Supporting material

Supporting material

Supporting material
