# Supplementary material for: Live birth/parity number and the risk of incident hypertension among parous women during over 13 years of follow‐up
Source: J Clin Hypertens (Greenwich). 2021 Oct 17;23(11):2000–8. doi: 10.1111/jch.14369 (PMC8630610; doi:10.1111/jch.14369)
Supplement: Supplementary file 1 — Supporting material [file JCH-23-2000-s003.docx]

| **Supplementary Table 1. Multivariable hazard ratios (HR) and 95% confidence intervals (CI) of incident hypertension according to the number of parity among women: Tehran Lipid and Glucose Study, Iran, 1999-2018.** | | | | | | | | |
| --- | --- | --- | --- | --- | --- | --- | --- | --- |
|  | **Model 1** | | **Model 2** | | **Model 3** | | **Model 4** | |
|  | **HR (95% CI)** | **P-value** | **HR (95% CI)** | **P-value** | **HR (95% CI)** | **P-value** | **HR (95% CI)** | **P-value** |
| **Number of parity** |  |  |  |  |  |  |  |  |
| - **1** | **1.01 (0.72-1.41)** | **0.951** | **1.00 (0.72-1.40)** | **0.999** | **1.00 (0.72-1.40)** | **0.982** | **0.99 (0.71-1.39)** | **0.970** |
| - **2** | **Reference** |  | **Reference** |  | **Reference** |  | **Reference** |  |
| - **3** | **1.37 (1.11-1.69)** | **0.003** | **1.31 (1.06-1.62)** | **0.014** | **1.30 (1.05-1.61)** | **0.016** | **1.25 (1.01-1.54)** | **0.043** |
| - **≥ 4** | **1.68 (1.36-2.07)** | **<0.001** | **1.43 (1.16-1.77)** | **0.001** | **1.40 (1.14-1.74)** | **0.002** | **1.40 (1.13-1.74)** | **0.002** |
| **Age (year)** | **1.05 (1.04-1.06)** | **<0.001** | **1.04 (1.03-1.06)** | **<0.001** | **1.05 (1.03-1.06)** | **<0.001** | **1.04 (1.03-1.05)** | **<0.001** |
| **BMI (kg/m^2^)** |  |  | **1.03 (1.01-1.06)** | **0.009** | **1.03 (1.01-1.06)** | **0.011** | **1.03 (1.00-1.05)** | **0.033** |
| **WC (cm)** |  |  | **1.01 (1.00-1.02)** | **0.020** | **1.01 (1.00-1.02)** | **0.027** | **1.01 (1.00-1.02)** | **0.150** |
| **DM** |  |  | **1.80 (1.46-2.22)** | **<0.001** | **1.72 (1.39-2.13)** | **<0.001** | **1.61 (1.30-1.99)** | **<0.001** |
| **Family history of**  **premature CVD** |  |  | **1.23 (1.05-1.45)** | **0.011** | **1.22 (1.04-1.44)** | **0.014** | **1.20 (1.02-1.41)** | **0.027** |
| **Current smoking** |  |  | **0.91 (0.67-1.26)** | **0.583** | **0.91 (0.66-1.25)** | **0.548** | **0.97 (0.70-1.33)** | **0.839** |
| **TG/HDL-C** |  |  | **1.01 (1.00-1.03)** | **0.140** | **1.01 (1.00-1.03)** | **0.124** | **1.01 (1.00-1.03)** | **0.128** |
| **Menopause** |  |  | **1.03 (0.84-1.27)** | **0.761** | **1.04 (0.84-1.27)** | **0.733** | **1.08 (0.87-1.32)** | **0.489** |
| **OCP use** |  |  | **1.05 (0.78-1.42)** | **0.754** | **1.04 (0.77-1.40)** | **0.814** | **1.02 (0.75-1.38)** | **0.892** |
| **History of preeclampsia** |  |  |  |  | **1.35 (1.04-1.76)** | **0.023** | **1.32 (1.02-1.71)** | **0.036** |
| **History of GDM** |  |  |  |  | **1.17 (0.97-1.42)** | **0.107** | **1.20 (0.99-1.45)** | **0.066** |
| **Prehypertension** |  |  |  |  |  |  | **2.30 (1.99-2.65)** | **<0.001** |
| **BMI: body mass index; WC: waist circumference; DM: diabetes mellitus; CVD: cardiovascular disease; FH: family history; TG: triglycerides; HDL-C:** **high-density lipoprotein cholesterol; OCP: oral contraceptive pill; GDM: gestational diabetes mellitus.**  **Model 1: adjusted for age.**  **Model 2: adjusted for age, BMI, WC, DM, family history of premature CVD, current smoking, TG/HDL-C, menopausal status, and OCP use.**  **Model 3: Model 2 + further adjusted for preeclampsia and GDM.**  **Model 4: Model 3 + further adjusted for prehypertension.** | | | | | | | | |
