# Supplementary material for: Live birth/parity number and the risk of incident hypertension among parous women during over 13 years of follow‐up
Source: J Clin Hypertens (Greenwich). 2021 Oct 17;23(11):2000–8. doi: 10.1111/jch.14369 (PMC8630610; doi:10.1111/jch.14369)
Supplement: Supplementary file 2 — Supporting material [file JCH-23-2000-s002.docx]

| **Supplementary Table 2. Multivariable hazard ratios (HR) and 95% confidence intervals (CI) of incident hypertension per additional parity among women: Tehran Lipid and Glucose Study, Iran, 1999-2018.** | | | | | | | | |
| --- | --- | --- | --- | --- | --- | --- | --- | --- |
|  | **Model 1** | | **Model 2** | | **Model 3** | | **Model 4** | |
|  | **HR (95% CI)** | **P-value** | **HR (95% CI)** | **P-value** | **HR (95% CI)** | **P-value** | **HR (95% CI)** | **P-value** |
| **Each additional parity** | **1.08 (1.04-1.12)** | **<0.001** | **1.06 (1.02-1.10)** | **0.004** | **1.06 (1.02-1.10)** | **0.006** | **1.06 (1.02-1.10)** | **0.004** |
| **Age (year)** | **1.05 (1.04-1.06)** | **<0.001** | **1.04 (1.03-1.06)** | **<0.001** | **1.05 (1.03-1.06)** | **<0.001** | **1.04 (1.03-1.05)** | **<0.001** |
| **BMI (kg/m^2^)** |  |  | **1.03 (1.01-1.06)** | **0.008** | **1.03 (1.01-1.06)** | **0.030** | **1.03 (1.00-1.05)** | **0.030** |
| **WC (cm)** |  |  | **1.01 (1.00-1.02)** | **0.014** | **1.01 (1.00-1.02)** | **0.020** | **1.01 (1.00-1.02)** | **0.119** |
| **DM** |  |  | **1.77 (1.44-2.19)** | **<0.001** | **1.70 (1.37-2.10)** | **<0.001** | **1.59 (1.29-1.96)** | **<0.001** |
| **Family history of**  **premature CVD** |  |  | **1.23 (1.05-1.45)** | **0.011** | **1.22 (1.04-1.43)** | **0.015** | **1.20 (1.02-1.41)** | **0.025** |
| **Current smoking** |  |  | **0.90 (0.65-1.23)** | **0.503** | **0.89 (0.65-1.22)** | **0.472** | **0.95 (0.69-1.31)** | **0.758** |
| **TG/HDL-C** |  |  | **1.01 (1.00-1.03)** | **0.097** | **1.01 (1.00-1.03)** | **0.086** | **1.01 (1.00-1.03)** | **0.092** |
| **Menopause** |  |  | **1.02 (0.83-1.25)** | **0.847** | **1.03 (0.83-1.26)** | **0.810** | **1.06 (0.86-1.31)** | **0.561** |
| **OCP use** |  |  | **1.05 (0.77-1.42)** | **0.753** | **1.04 (0.77-1.40)** | **0.821** | **1.02 (0.75-1.37)** | **0.921** |
| **History of preeclampsia** |  |  |  |  | **1.36 (1.05-1.77)** | **0.020** | **1.33 (1.03-1.73)** | **0.031** |
| **History of GDM** |  |  |  |  | **1.19 (0.98-1.44)** | **0.077** | **1.21 (1.00-1.47)** | **0.045** |
| **Prehypertension** |  |  |  |  |  |  | **2.31 (2.00-2.66)** | **<0.001** |
| **BMI: body mass index; WC: waist circumference; DM: diabetes mellitus; CVD: cardiovascular disease; FH: family history; TG: triglycerides; HDL-C:** **high-density lipoprotein cholesterol; OCP: oral contraceptive pill; GDM: gestational diabetes mellitus.**  **Model 1: adjusted for age.**  **Model 2: adjusted for age, BMI, WC, DM, family history of premature CVD, current smoking, TG/HDL-C, menopausal status, and OCP use.**  **Model 3: Model 2 + further adjusted for preeclampsia and GDM.**  **Model 4: Model 3 + further adjusted for prehypertension.** | | | | | | | | |
