# Supplementary material for: Live birth/parity number and the risk of incident hypertension among parous women during over 13 years of follow‐up
Source: J Clin Hypertens (Greenwich). 2021 Oct 17;23(11):2000–8. doi: 10.1111/jch.14369 (PMC8630610; doi:10.1111/jch.14369)
Supplement: Supplementary file 3 — Supporting material [file JCH-23-2000-s001.docx]

| **Supplementary Table 3. Multivariable hazard ratios (HR) and 95% confidence intervals (CI) of incident hypertension among women without having other delivery after baseline (n: 2055): Tehran Lipid and Glucose Study, Iran, 1999-2018.** | | | | | | | | |
| --- | --- | --- | --- | --- | --- | --- | --- | --- |
|  | **Model 1** | | **Model 2** | | **Model 3** | | **Model 4** | |
|  | **HR (95% CI)** | **P-value** | **HR (95% CI)** | **P-value** | **HR (95% CI)** | **P-value** | **HR (95% CI)** | **P-value** |
| **Each additional live birth** | **1.08 (1.04-1.13)** | **<0.001** | **1.06 (1.02-1.11)** | **0.005** | **1.06 (1.02-1.11)** | **0.008** | **1.06 (1.02-1.11)** | **0.006** |
|  |  |  |  |  |  |  |  |  |
| **Number of live birth** |  |  |  |  |  |  |  |  |
| - **1** | **1.05 (0.74-1.50)** | **0.773** | **1.01 (0.71-1.44)** | **0.940** | **1.02 (0.72-1.45)** | **0.917** | **0.99 (0.70-1.41)** | **0.965** |
| - **2** | **Reference** |  | **Reference** |  | **Reference** |  | **Reference** |  |
| - **3** | **1.36 (1.10-1.68)** | **0.004** | **1.29 (1.05-1.60)** | **0.018** | **1.28 (1.04-1.58)** | **0.022** | **1.24 (1.00-1.53)** | **0.052** |
| - **≥ 4** | **1.65 (1.34-2.04)** | **<0.001** | **1.41 (1.14-1.74)** | **0.002** | **1.38 (1.12-1.72)** | **0.003** | **1.39 (1.12-1.72)** | **0.003** |
|  |  |  |  |  |  |  |  |  |
| **Each additional parity** | **1.08 (1.04-1.12)** | **<0.001** | **1.06 (1.02-1.10)** | **0.007** | **1.05 (1.01-1.10)** | **0.011** | **1.06 (1.01-1.10)** | **0.008** |
|  |  |  |  |  |  |  |  |  |
| **Number of parity** |  |  |  |  |  |  |  |  |
| - **1** | **1.04 (0.72-1.51)** | **0.815** | **1.00 (0.69-1.44)** | **0.995** | **1.01 (0.70-1.45)** | **0.974** | **1.00 (0.70-1.45)** | **0.980** |
| - **2** | **Reference** |  | **Reference** |  | **Reference** |  | **Reference** |  |
| - **3** | **1.37 (1.11-1.70)** | **0.004** | **1.31 (1.05-1.62)** | **0.014** | **1.30 (1.05-1.61)** | **0.017** | **1.25 (1.01-1.55)** | **0.043** |
| - **≥ 4** | **1.68 (1.36-2.08)** | **<0.001** | **1.43 (1.16-1.78)** | **0.001** | **1.41 (1.13-1.75)** | **0.002** | **1.42 (1.14-1.76)** | **0.002** |
| **Model 1 is adjusted for age.**  **Model 2 is adjusted for age, body mass index, waist circumference, diabetes mellitus, family history of premature cardiovascular disease, current smoking, triglycerides/ high-density lipoprotein cholesterol, menopausal status, and oral contraceptive pill (OCP) use.**  **Model 3: Model 2 + further adjusted for preeclampsia and gestational diabetes mellitus.**  **Model 4: Model 3 + further adjusted for prehypertension.** | | | | | | | | |
